# Gibson and Pictures in Perspective: Reverse the Directions

**DOI:** 10.1177/20416695211047259

**Published:** 2021-10-19

**Authors:** John M. Kennedy

**Affiliations:** University of Toronto Scarborough, Toronto, Canada

**Keywords:** 3D perception, blindness, contours/surfaces, depth, spatial vision, shapes/objects, scene perception, pointing/hitting, perception, multisensory/cross-modal processing

## Abstract

In his extensive writing about pictures, James J. Gibson offered perspective formulae for square tiles projecting trapezoids onto a picture plane, foreshortening to zero height with distance. I reverse the claim: as distance decreases, the trapezoids increase to infinite height, in marginal distortion, or forelengthening. I also reverse the direction of projection. Usually considered to be incoming, from the distant tile to the picture plane, in reverse—outgoing—the tiles have directions from the center of projection, with implications for haptics and people who are blind. A drawing of a cube illustrates the argument. It is by an adult who is blind. It includes foreshortening, and shows directions of surfaces from a vantage point.

## Gibson and Pictures in Perspective: Reversing the Projection

Gibson (1950, 1979) wrote extensively about pictures, theorizing about the natural world and artifice, outline, and projection and foreshortening. I suggest here that his linear and quadratic formulae for foreshortening can produce forelengthening, and apply to directions from vantage points in touch. The directions may be entertained by people creating a picture, including people who are visually handicapped. I provide an example from an adult who is blind.

### Personal Note

Gibson supervised my doctoral thesis on outline drawings, figure-ground, and perspective (Kennedy, 1971, 1974), which argued that lines stand for visible discontinuities, most notably edges of surfaces, offering vivid perception of depth and slant.^1^ Lines can also show the shapes of shadows, highlights, and color patches, but the shapes do not darken, brighten or become colored (Casati & Cavanagh, 2019; Kennedy, 1993).

Besides being a giant in perception theory, Gibson was also a down-to-earth, enthusiastic, and attentive supervisor. Late in the afternoon, I would give him drafts of my thesis. Comments arrived breakfast-time the next day!

### Gibson in 1950

**Figure 1.**
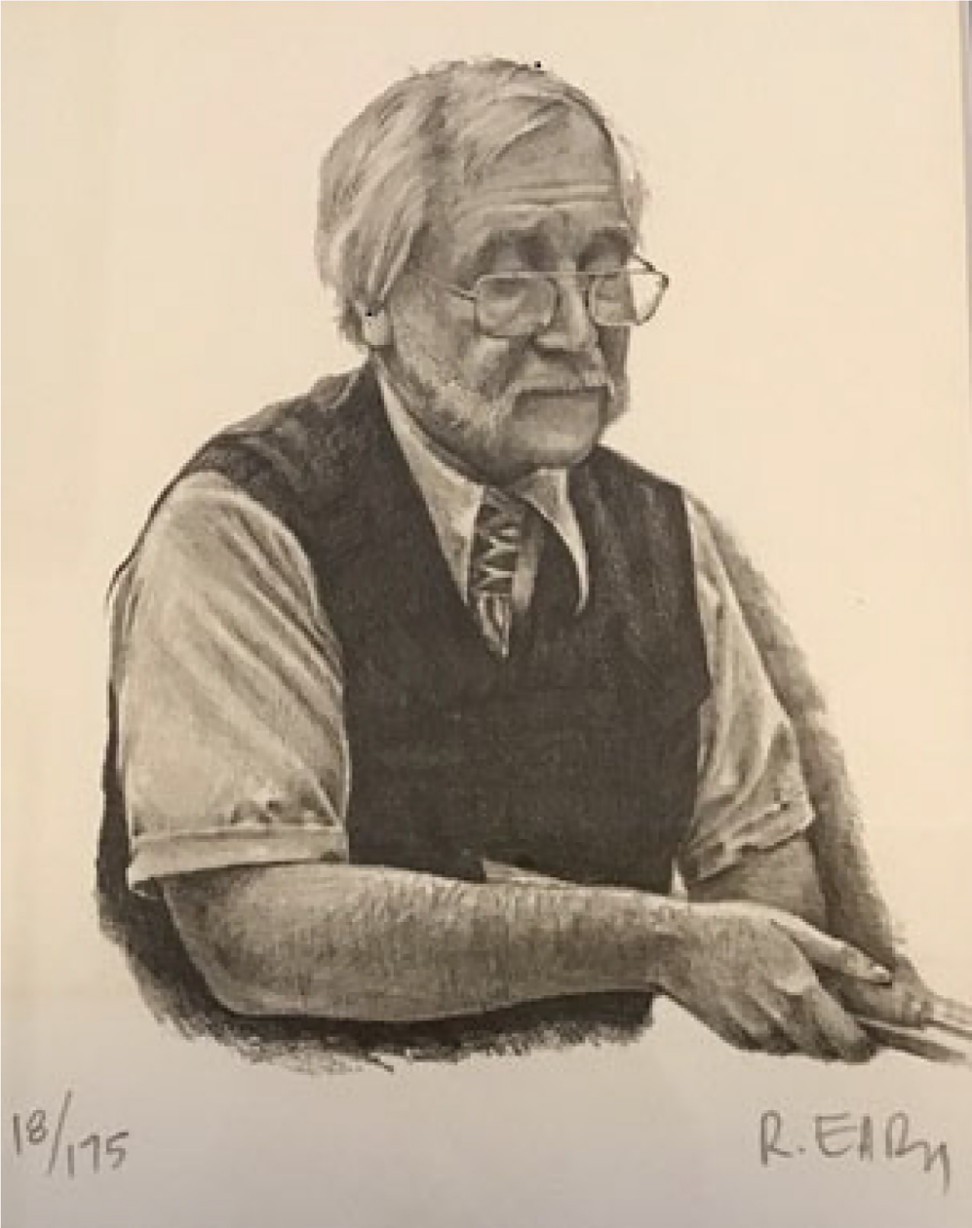
Realistic drawing of an art teacher, by Ron Eady, Canadian artist, with textures and shadows. Copy of print 18 of 175. Published here by permission of Ron Eady.

**Figure 2.**
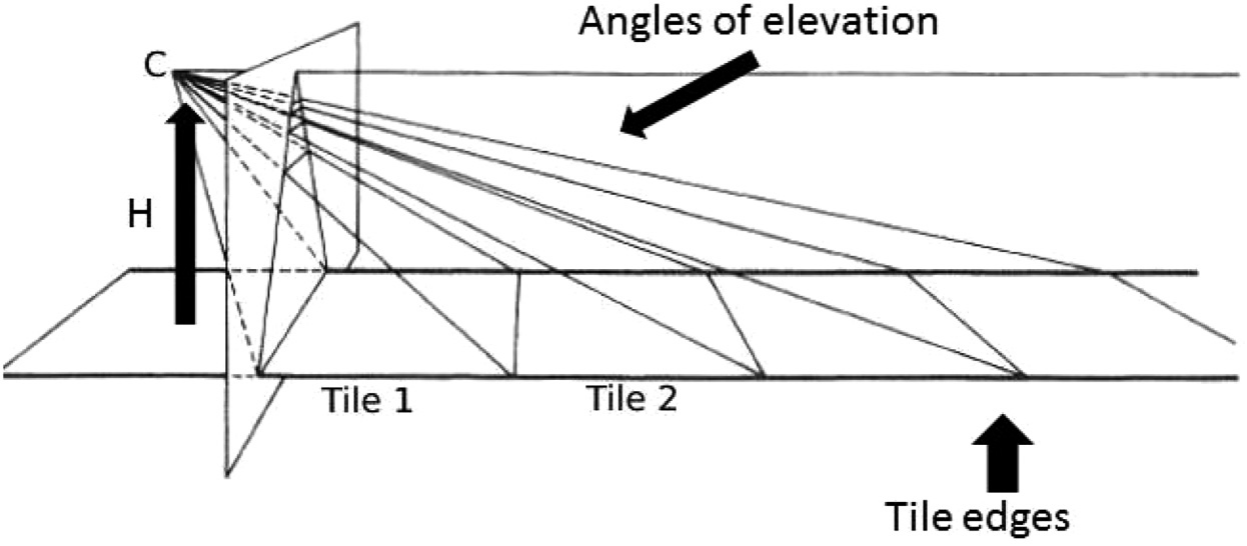
From Gibson (1979, p. 265), his Figure 15.2 with text added by Selene Carboni; image used with her permission. On the vertical picture plane, square Tiles 1, 2, etc. are depicted by foreshortened trapezoids. Edges of the tiles are shown by lines. C is the center of projection, height *H*. *Azimuth* is parallel to the picture plane. Obliques show angles of elevation. The tile edge shared by Tile 1 and Tile 2 is at an *elevation* from the center of projection of about 45° from vertical. The angle subtended by the *azimuth extent* of a tile diminishes at rate *D* with distance and the *elevation extent* by *D*^2^. If the center of projection is raised, forelengthening results. Tile 1 has an edge in contact with the picture plane. Its size is constant as *C* increases in height and the trapezoid depicting Tile 1 becomes forelengthened.

Gibson (1950) asked what allows perception of the natural world. He included realistic pictures with shading and texture, much like Figure 1, and he accompanied illustrations of foreshortening (Figure 2) with linear perspective drawings of ground plains, dotted with horizontal and vertical lines.

### What is a Picture?

Gibson's argument about optic information and pictures ran as follows: In an environment made of illuminated textured surfaces, surrounded by air, light converges in keeping with linear perspective to a center of projection as shown in Figure 2. The optic array at the center specifies the surrounding world. Pictures sample from the informative features of natural light, including ratios and other formless invariants. He commented, “A frozen form does not specify the solid shape of an object, only some of the invariant features that a solid object must have” (Gibson, 1979, p. 257).

In natural light, Gibson argued, there is information for size and distance. Optic texture gradients specify surfaces receding from the vantage point. At a center of projection, height *H*, if the top of an object standing on the ground is pictured as reaching the horizon, its height is *H*, and more generally, “the ratio of the height of the object to the height of the point of observation is approximately equal to the ratio of the optic array angle subtended vertically by the object to the optic array angle subtended vertically by the portion of the object that is below the horizon” (Sedgwick, 1973, p. 10). Further, by isosceles triangles, a spot on the ground 45° from the vantage point is *H* distance along the ground (Wnuczko & Kennedy, 2021).

Gibson's argument, foreshadowed by Alhazen and Piero della Francesca, undercut Bishop Berkeley's position that light at a point of observation has no distance information, only direction. Reliable optic information renders needless the solipsism that we think we are living in a world but we are actually only living in our own minds since perception is mostly imagination (perhaps even *imagining that we are imagining*, on a slippery slope into an infinite regress).

That information is accessed by perception finesses the need to create a mental world of memories and guesses as a solution to the inverse problem of getting from a sense's input all the way back to the source of the sensory information. Simply put, Gibson championed natural laws, notably linear perspective, governing the relationship between observer and observed.

Informative light is created by the causal structure of the natural universe, at many scales, Gibson pointed out, to his great credit (Dretske, 1981). In contrast, pictures like Figure 1, which uses accurate projection and enough detailed shape and chiaroscuro (light and shade) to be specific to an individual at a time in his life, are artificial. Pictures with this degree of specificity are not found in nature, on land, sea, or sky. Rare landscapes such as Cave Hill, Northern Ireland, (dubbed Napoleon's Nose) and a cliff at St. Kilda's, Scotland, and a rock in Wales (Pitt's Head) resemble human profiles from some vantage points, but the resemblance has low fidelity compared to Figure 1. In Iceland and Italy, celebrated rocks resemble elephants, but only to a degree. A rock resembles the head of a bear in *Islands: Canoe Lake* (1916; Cockburn, 2020), a well-known painting by Tom Thomson, doyen of Canadian art, but though it is unmistakable once pointed out, the resemblance is modest. A true likeness, one with high fidelity, is something created. Shadows, like silhouettes, only offer a fraction of the features of Figure 1. If Figure 1 is 9/10 on a fidelity scale, the rocks and landscapes mentioned here might be rated just 1 or 2 or 3.

### Perspective and the Direction of Projection

Cast shadows, befitting linear perspective, are short when the sun is high, and long when it is low. The first case is foreshortening and the second might be called forelengthening. Evidently, projection offers two possibilities. Here, I point out that Gibson's formulae can be shown to embrace both.

Like his friend and colleague Sir Ernst Gombrich, art historian, Gibson rejected the idea that pictures in linear perspective are a convention (Feeney, 2019; Goodman, 1968). Rather, they fit with the behavior of light. In foreshortening, the angles subtended by the azimuths of tiles lying on the ground diminish at rate *D* with distance. In contrast, the angle subtended by the tile's extent in depth diminishes at rate *D*^2^ (Figure 2). Gibson wrote, “The squares of the track correspond to trapezoids on the picture plane, diminishing as a function of distance” (Gibson, 1979, p. 265). Let us add here that the claim is reversible: The trapezoids also increase as distance decreases. Consider the implications.

**Figure 3.**
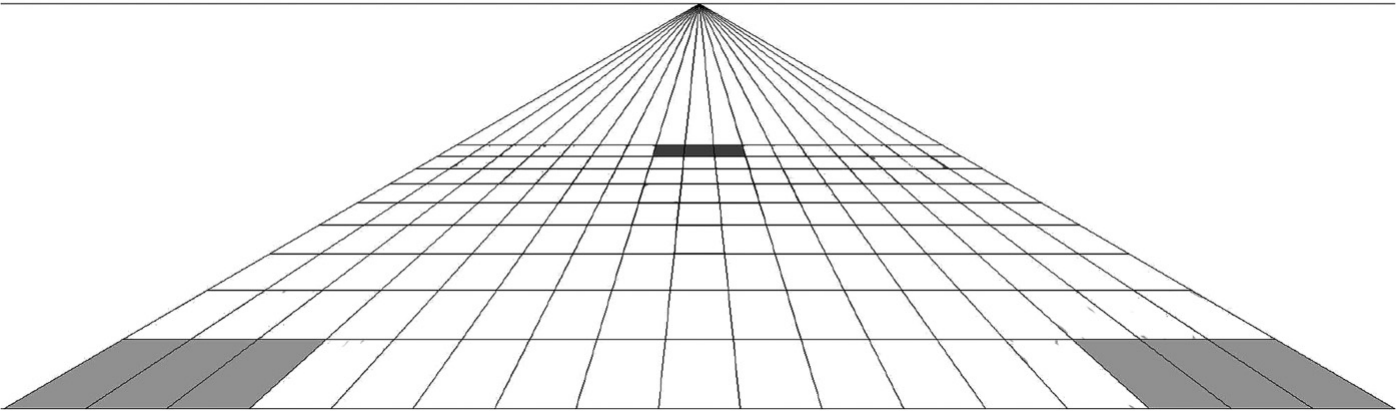
A wide-angle linear perspective picture of square tiles on a piazza. By Selene Carboni, with permission. Tiles higher in elevation (such as the dark tiles) are foreshortened. Lower tiles are forelengthened, marginal gray tiles most obviously, and tiles on the sides are also sheared.

In Figure 3, trapezoids forelengthen in what Kubovy (1986) called marginal distortion (Kennedy & Juricevic, 2002; Pirenne, 1970, p. 127). Why? Again, shadows may help reveal the answer. If the sun's elevation is 45° from vertical (declination also 45° naturally), our shadow on a flat ground is our height, by right-angled isosceles triangles. Likewise, from the center of projection in Figure 2, Tile 1 (dimensions 1 × 1, touching the picture plane) has an edge elevated 45°. By isosceles triangles, it projects a trapezoid of height 1.

If the height *H* of the center of projection increases, Tile 1 projects a taller trapezoid. Let us say *H* is 1.5. If the center of projection were to move up to *H* 3.0, the edge of the tile would have elevation 27°, and its height 2, double its azimuth, and forelengthened.

If *H* increases to 4.5, the edge would have elevation 18°, and the trapezoid's height would be 3. Further, if the edge had 10° elevation, the trapezoid would have a height of 6*H*.

Besides foreshortening to zero height as their distance increases—Gibson's point—his formulae allow the trapezoids to forelengthen to infinity as *H* increases. The operative function is *H* in proportion to tile size, which controls the angle of elevation. Although *D*^2^ can shorten Figure 2’s trapezoids for tiles at long distances—multiples of the 1 × 1 of Tile 1—perforce it elongates them for short distances, less than 1.

Elevation 45° divides forelengthening from foreshortening (Juricevic & Kennedy, 2006; Kennedy & Juricevic, 2002; Kubovy, 1986). In Figure 3, tiles with high elevation foreshorten and lower tiles forelengthen.

Forelengthening appears in photos taken with a wide-angle lens, in news shots in periodicals for example. Traffic-camera videos use a wide-angle lens. In traffic reports on TV long trucks often appear realistic in the center of view and to stretch—be magnified—as they pass to the screen's periphery, in marginal distortion. If the observer views from the proper center of projection (close to the screen) the trucks appear rigid. Being too close minifies the trucks.

To avoid possible confusion, I should note that in addition to being at improper distances from the screen, the observer can be to one side of the center of projection. The result is shearing*.* Viewing from the side has distinct effects. Eyes in portraits follow the observer—perhaps eerily—in the Mona Lisa Effect (Hecht et al., 2014). Likewise, viewed from the side, roads running directly away from the observer maintain that direction faithfully, one might say, but roads parallel to the picture plane stubbornly stay parallel—the differential-rotation effect (Goldstein, 1987). The effects fit affine transformations (Cutting, 1988; Farber & Rosinski, 1978; Sedgwick, 1991). In sum, centres of projection sit apart from the picture plane and are surrounded by alternative points of observation—at other distances or lateral locations. Putting oneself close to the center of projection is customary, a kind of *viewing* convention one might say. Departing more and more from the center increases the risk that optic information for perceptual constancy is missed or ineffective. Be that as it may, a customary viewpoint—a viewing convention—is a far cry from the idea that perspective has no basis in optics and perception and is merely a conventional tactic in *constructing* a picture, the idea that Gibson and Gombrich dismissed.

Kubovy (1986) described shape constancy failing when a wide-angle picture is “viewed from somewhere other than the center of projection” (p. 52). In Figure 3, the center of projection is close to the image (distant from it by under half the picture's width). Readers will normally be viewing Figure 3 from farther, and lower tiles will appear stretched.

**Figure 4.**
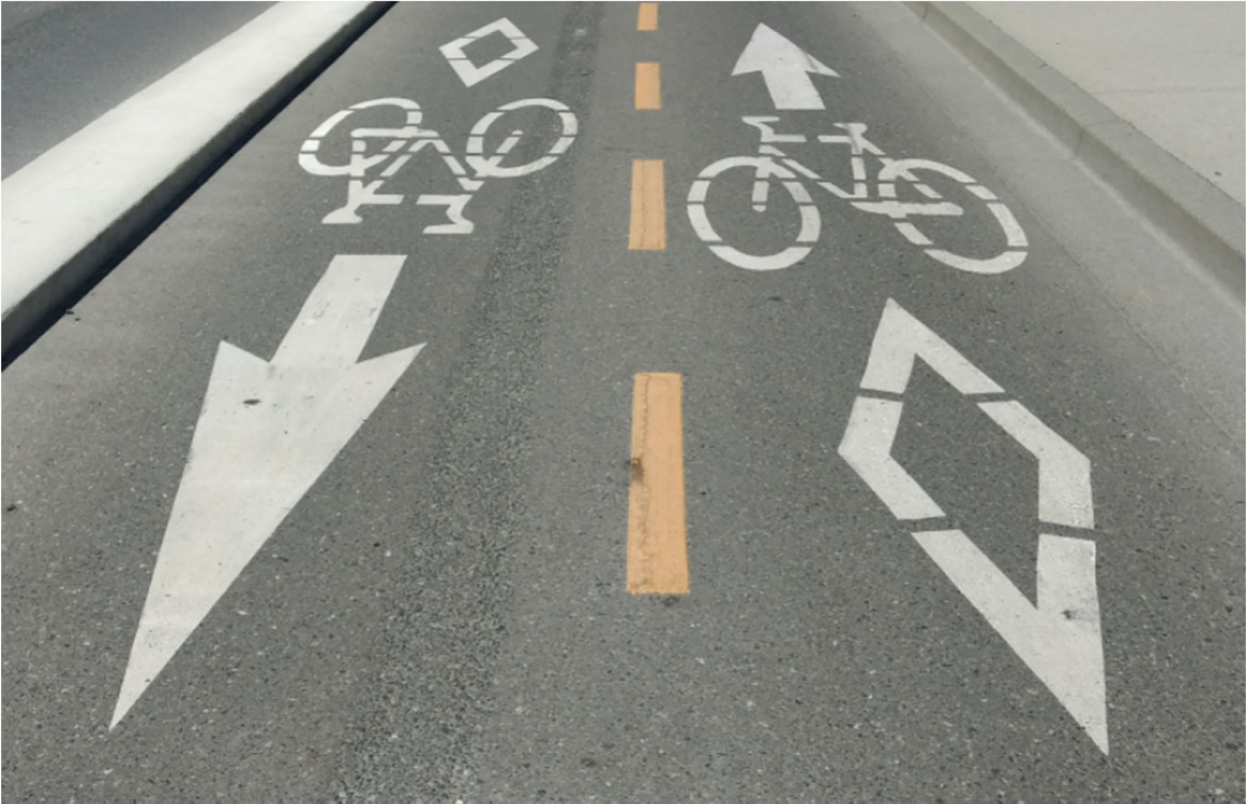
Bikes, arrows, and a diamond painted on a bike path and foreshortened with distance. Twin forms may appear to be physically different in the scene. The arrowhead on the left may appear sharper. See also Wnuczko and Kennedy (2021).

Azimuth extents shrink at rate *D*, elevation extents at *D*^2^. Figure 4 shows the results for arrowheads on a path. The arrow on the right is more distant and at an extreme optic slant, and is highly foreshortened as a result. It looks blunt. That on the left, a twin arrow, is closer, at a lesser slant, and looks sharper. Perception appreciates linear perspective to some extent, as Gibson suggested, but as observation of Figures 3 and 4 indicates, it does not quite keep up with the difference between *D* and *D*^2^ rates (Wnuczko & Kennedy, 2021; Wnuczko et al., 2016).

A picture is a frozen moment, in Gibson's terms. If a movie camera taking the photo for Figure 4 was walked forward toward the arrowheads, motion would yield more optic information, and greater constancy would be achieved (Wang et al., 2020).

### Gibson, Gombrich, and Projection Reversed

About Figure 2, Gibson (1979) wrote “The parallel edges of a track project to straight lines” (p. 265). In Durer prints, a point on an object is strung to an observer's vantage point (Gombrich, 1961, p. 251, 306). Durer shows one can “draw straight lines to that [vantage] point from any part of the object's surface” (Gombrich, 1961, p. 250). But strings and lines are not arrows. Rather, they connect two ways. Besides light projecting to the vantage point, the lines show the directions of points in the scene from the observer.

Instead of considering light coming toward them, observers drawing a scene may envisage the directions of an object from where they are stationed. A dot on the picture plane in that direction would occlude the point in the scene. From their point of regard, artists may ask themselves what is to the left, right, above, and below the item in the scene. That is, in practice, the goal for someone drawing a scene can be to consider where are things from here*,* not how would this scene project to the picture plane.

Directions going outward may often be what people envisage when drawing. Reversing the direction of the arrow of projection (Kubovy, 1986) from in-coming to out-directed may be helpful for theory of perspective drawings by novices and people who are blind.

Edges and corners are tangible. So too is shape. Observers have vantage points in touch as well as vision. We reach out to left, right, up, and down to touch objects arrayed to our left, our right, above us, and below us. Ergo, drawing with some perspective principles may be available to people who are blind as well as those who are sighted (Heller et al., 1996; Kennedy, 1993, 2019; Wnuczko & Kennedy, 2014).

**Figure 5.**
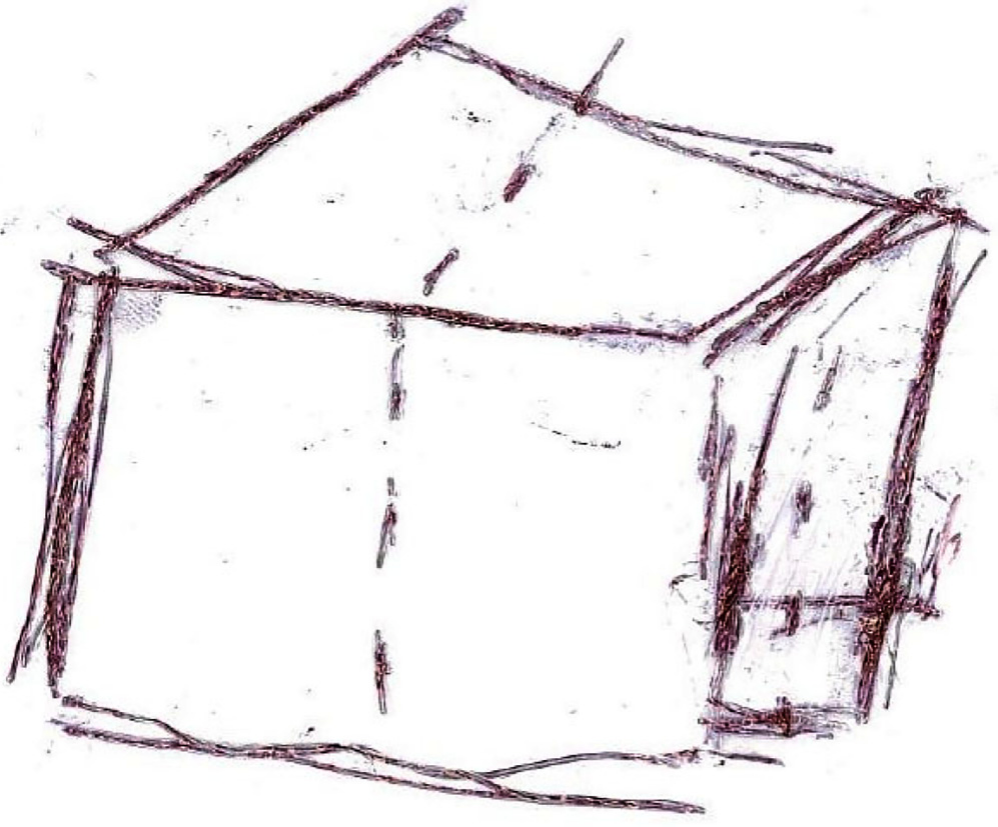
Raised-line drawing of a cube by M, a woman in her 30s who has been blind since infancy. She had some experience drawing as a teenager. From Carboni et al. (2021), with permission. Dotted lines show the middles of faces, M said. Of interest, the top and side are foreshortened.

Figure 5 (Carboni et al., 2021) is a drawing of a cube by a woman, M, who is blind, with modest experience drawing. The side and top of the cube are foreshortened as in drawings by sighted children aged about 9–12 years, and many sighted adults (Chen, 1985; Howard & Allison, 2011; Mitchelmore, 1985). The rough lines are tokens for straight lines (types) depicting the edges of the cube surfaces (Willats, 1985, 2005). The Y junction shows a cubic-corner, in accord with Perkins Laws (Kubovy, 1986).

M may have asked herself what faces of the cube would be evident in reaching out from her station point. What corners would be touched? How would the top and side be available? In matters of direction, how would they differ from the front? The elevations of the near and rear edges of the top would be close in direction. The azimuth directions of the near and far corners of the side to the right would also be close. The result is foreshortening. These considerations could lead M to draw three faces around a Y junction, with two faces slim.

(M was born at 7 months, premature, and placed in an incubator. As an infant, she had severe low vision, left eye acuity 20/400 (N.B. 20/200 is “legally blind.”). Her right eye was totally blind. At age 20 years, the left-eye retina detached. She is now only light sensitive. At school, M used video magnifiers to read, study text, and to draw occasionally. She made this raised-line sketch in a drawing class organized by her commune. That she gave permission to publish her drawing is very much appreciated. See also Carboni et al., 2021.

To conclude, Gibson offered formulae for perspective. They generate foreshortening and forelengthening in pictures. They apply to light coming to a center of projection and to directions from our vantage point in vision and touch. The directions may be a basis for drawing with some perspective effects, in sketches made by novices and people with visual impairments.

## Notes

1. Gibson wrote about pictures for 50 years, 1929–1979. He picked out major topics—outline, surfaces, projection, and specification. In his graduate seminar, he used my thesis on pictures for several years as an introduction to his ideas, Patrick Cabe told me. Here I am also 50 years on (Carboni et al., 2021), passing on his interests. A giant in the history of perception, he was also friendly and enthusiastic, stimulating and profound. Rotating a stick in water about its axis leaves its profile constant—as Albert Yonas demonstrated to us—and I reported to Gibson that children aged 5–7 years, seeing this, then judge “It's straight!” (Gibson et al., 1967). Gibson queried “What do you mean by straight?” I went rather quiet! I went away and came back with 5 or so answers. In 1976, Gibson, visiting my home, came in asserting blind people could not understand pictures. Before he left he said “Of course blind people are able to understand pictures!” His ability to think through arguments anew and to reject an old position has been important to me. I like to tell my students “This idea, made up by John Kennedy, has the advantage of being testable, and the disadvantage of being false.” We had interests in common. Both of us fenced at college, and spent time on the boards, acting in plays at Cornell. Meals together included Thanksgiving, lunches with visiting speakers, and dinners at conferences talking to Rudolf Arnheim, Sir Ernst Gombrich, Nelson Goodman, Gunnar Johannson, Julian Hochberg, Fabio Metelli, and David Krech, to name a few. A man of style and substance, much missed.
